# Co-diversification of an intestinal *Mycoplasma* and its salmonid host

**DOI:** 10.1038/s41396-023-01379-z

**Published:** 2023-02-17

**Authors:** Jacob A. Rasmussen, Pia Kiilerich, Abdullah S. Madhun, Rune Waagbø, Erik-Jan R. Lock, Lise Madsen, M. Thomas P. Gilbert, Karsten Kristiansen, Morten T. Limborg

**Affiliations:** 1 Laboratory of Genomics and Molecular Biomedicine, Department of Biology, University of Copenhagen, Copenhagen, Denmark; 2 Center for Evolutionary Hologenomics, GLOBE Institute, Faculty of Health and Medical Sciences, University of Copenhagen, Copenhagen, Denmark; 3 Danish Center for Neonatal Screening, Department of Congenital Disorders, Statens Serum Institut, 2300 Copenhagen, Denmark; 4 Institute of Marine Research, Bergen, Norway; 5 Department of Natural History, NTNU University Museum, Norwegian University of Science and Technology (NTNU), Trondheim, Norway; 6 Institute of Metagenomics, Qingdao-Europe Advanced Institute for Life Sciences, Qingdao, China

**Keywords:** Metagenomics, Population genetics, Microbiome, Molecular evolution, Bacterial genetics

## Abstract

Understanding the evolutionary relationships between a host and its intestinal resident bacteria can transform how we understand adaptive phenotypic traits. The interplay between hosts and their resident bacteria inevitably affects the intestinal environment and, thereby, the living conditions of both the host and the microbiota. Thereby this co-existence likely influences the fitness of both bacteria and host. Whether this co-existence leads to evolutionary co-diversification in animals is largely unexplored, mainly due to the complexity of the environment and microbial communities and the often low host selection. We present the gut metagenome from wild Atlantic salmon (*Salmo salar*), a new wild organism model with an intestinal microbiota of low complexity and a well-described population structure, making it well-suited for investigating co-evolution. Our data reveal a strong host selection of a core gut microbiota dominated by a single *Mycoplasma* species. We found a clear co-diversification between the population structure of Atlantic salmon and nucleotide variability of the intestinal *Mycoplasma* populations conforming to expectations from co-evolution between host and resident bacteria. Our results show that the stable microbiota of Atlantic salmon has evolved with its salmonid host populations while potentially providing adaptive traits to the salmon host populations, including defence mechanisms, biosynthesis of essential amino acids, and metabolism of B vitamins. We highlight Atlantic salmon as a novel model for studying co-evolution between vertebrate hosts and their resident bacteria.

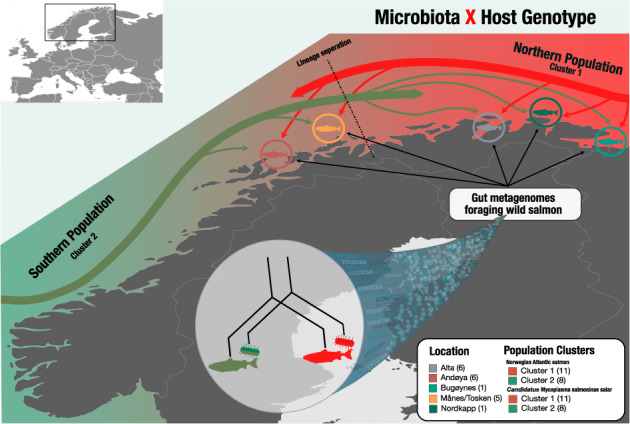

## Introduction

Co-evolutionary interactions between animals and their microbiota have become a key topic in evolutionary biology because of their adaptive relevance for understanding basic eco-evolutionary processes [1]. However, vertebrates and their associated microbial communities have yet to be systematically studied. One major drawback to understanding evolutionary host-microbiota relationships in many vertebrates, such as mammals, is that they are confounded by the high complexity of their microbial communities, challenging the detection of specific host-microbe interactions [2]. Dynamics in these complex systems are also often shaped by environmental factors limiting the ability to answer basic questions about broader evolutionary trends between host and resident bacteria, such as the simultaneous diversification of both host and microbe, which is often referred to as co-diversification [3].

One way to overcome these limitations is to investigate microbiotas of low diversity related to a host with a well-known population structure [3]. Teleosts offer an attractive option as more than 33,000 described species represent nearly half of all vertebrate species. Many teleosts have well-characterised population structures due to their commercial, cultural, and recreational importance [4]. However, despite representing the largest vertebrate group, very few host–bacteria systems have been investigated in teleosts compared to terrestrial species, such as hibernating brown bears [5], plant-eating rodents [6], and production animals such as pigs [7] and ruminants [8, 9]. Due to its significant commercial, cultural, and recreational importance, the Atlantic salmon (*Salmo salar*) provides a well-studied model system. Atlantic salmon has a well-described genome, evolutionary history, and population structure throughout its North Atlantic distribution [10–12]. These assets make Atlantic salmon an ideal model for studying general evolutionary aspects of host-microbe interactions in vertebrates.

Adult salmon are piscivorous, and their associated microbiota is thus expected to be relatively simpler than microbiomes originating from fish being omnivorous and herbivorous [13]. Recent surveys of intestinal microbial communities in salmonid species have revealed a general dominance of a single unique *Mycoplasma* species suggesting a close relationship with the salmon host [14–16]. Salmonid-related *Mycoplasmas* are hypothesised to be vertically transmitted between generations since i) they have not yet been discovered in the environment [17], ii) are prominent in both wild and farmed salmon [15–18], and iii) do not follow neutral processes in both wild and farmed individuals [16]. This consistent trend of intestinal microbial communities characterised by a low diversity makes Atlantic salmon a practical model for studying co-evolutionary relationships between vertebrate hosts and their core bacteria [19, 20].

Genome-resolved metagenomics offers a detailed resolution of microbial communities and microbial species. However, the often-reported low biomass of intestinal microbiota in salmonids has limited the broader application of shotgun metagenomic sequencing in these species [14, 15]. Therefore, previous studies have yet to use detailed genome-resolved metagenomics to uncover the functional and genomic legacy of the intestinal microbiome in wild populations of Atlantic salmon. Here, we use genome-resolved metagenomics and comparative genomics to investigate the metagenomic dynamics and hologenome of more than 70 adult foraging wild Atlantic salmon. We further compare the intestinal microbiota from Atlantic salmon with gut bacteria of other sympatric teleost species to elucidate environmental and host-related dynamics. Our study presents the first genome-resolved metagenome from wild Atlantic salmon, extending decades of PCR-based gene surveys, and highlights the value of applying a hologenomics approach [3] to study how host-microbiota relationships shape adaptive evolutionary processes.

## Results

**Fig. 1:**
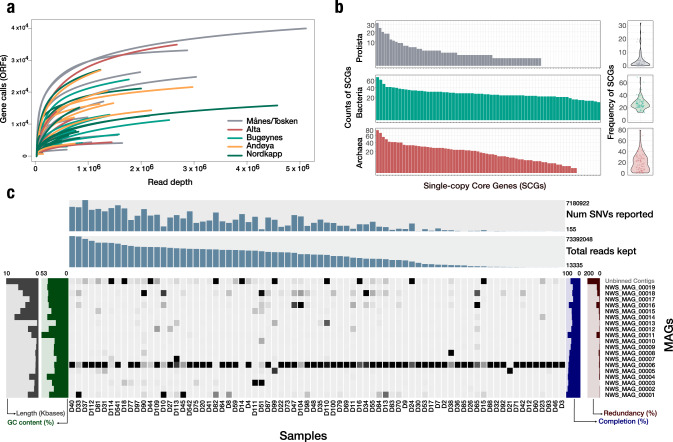
Overview of the Atlantic salmon metagenome. **a** Rarefaction curves of gene calls across all samples from the co-assembled metagenome. **b** Single-copy core gene estimates present protists, bacteria, and archaea. **c** Coverage heatmap of recovered MAGs from the gut microbiota of Atlantic salmon. The top barplot reports the number of single nucleotide variants (SNVs) reported per sample, and the bar plot below reports read depth per sample. From the left, grey bars indicate the log10 length of each MAG and unbinned contigs, green bars indicate GC content (%) per MAG, dark grey bars show the length of MAGs (kbases), and the heatmap of the MAGs is based on mean coverage per sample. White indicates low coverage, whereas black indicates high coverage. The heatmap is normalised according to the highest coverage of the most dominant MAG across each sample. Blue bars indicate completion (%) of each MAG, whereas burgundy indicates redundancy (%).

Metagenomes were recovered from 75 wild Atlantic salmon individuals from five different regions in northern Norway, including Alta, Andaøya, Bugøynes, Månes/Torsken, and Nordkapp, spanning more than 700 km across the North Atlantic Ocean. Metagenomic sequencing of all individuals resulted in 5806 million reads. Re-mapping of the host genome revealed that 53.8% (SD ± 27.7%) of the reads mapped to the host genome, a percentage lower than in previous studies [14, 15]. Removal of low-quality and non-microbial data resulted in a recovery of 40.7% (SD ± 27.8%) of the reads (Suppl. Table S1, Suppl. Fig. S1). We identified 2,241,791 non-redundant genes in scaffolds longer than 1000 nucleotides. Investigation of sequencing depth and gene calls’ rarefaction indicated sufficient metagenomic data saturation for a representative metagenomic analysis (Fig. 1a). Automatic binning and manual curation of co-assembly resulted in 19 non-redundant metagenome-assembled genomes (MAGs) containing 50,153,577 bases; see the summary of MAGs (Suppl. Table S2). The MAG catalogue represented 99.9% of the size-normalised bacterial data. Counting 71 bacterial single-copy core genes (SCGs) across the metagenome revealed that 23 bacterial genomes were potentially present in the metagenome, indicating that we recovered the majority of the MAGs (82.6 %) and that the metagenome of wild Atlantic salmon is characterised by a relatively low diversity (Fig. 1b, c).

### Genome-resolved metagenomics reveals a low diversity gut microbiota of the Atlantic salmon, indicating strong host selection

**Fig. 2:**
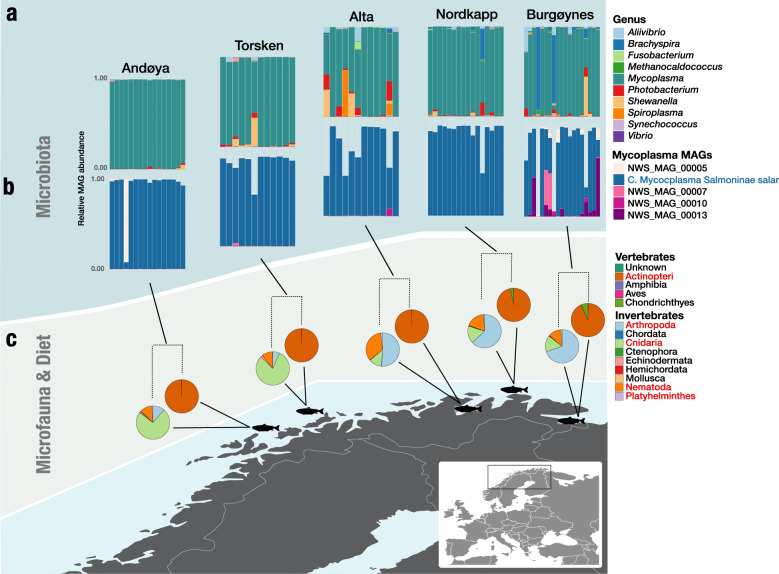
Overview of gut microbiota and microfauna composition of wild Atlantic salmon. Atlantic salmon were sampled across five locations in northern Norway, including Andøye, Torsken, Alta, Nordkapp, and Bugøynes, as indicated by black fish images. **a** illustrates the relative MAG abundance of all 19 MAGs, including all genera, and **b** focuses on the relative MAG abundance within the *Mycoplasma* genus. **c** Pie charts indicate microfauna and diet composition. The right pie chart indicates vertebrate composition, and the left indicates invertebrate composition.

Diversity analysis of MAGs revealed a gut microbiome of low diversity during the marine foraging life stage. Phylogenomics of recovered MAGs together with 3,207 bacterial reference genomes resulted in the taxonomic placement of MAGs, which included especially Tenericutes and Proteobacteria, but also Spirochaete, Cyanobacteria, and Pseudomonadota (Suppl. Fig. S2). Our analysis revealed no significant differences in microbiota composition between sampling locations (Fig. 2a, b, Suppl. Fig. S3). *Mycoplasma* was highly dominant (90.4% of the MAG profiled reads), consistent with previous findings [14, 15, 17, 18, 21, 22]. We found an intermittent high abundance of *Photobacterium phosphoreum*, a species of *Brachyspira* (formerly classified as *Brevinema andersonii*), and *Shewanella* MAGs across eight samples (Fig. 2a). Subsequently, we recovered low amounts of *Synechococcus, Vibrio, Aliivibrio salmonicida*, and *Methanocaldococcus* across the 75 Atlantic salmon.

A large proportion of the data represented *Mycoplasma*, which dominated overall and represented over 90.4 % of all host-filtered reads. Of the 19 MAGs recovered, five MAGs were within the family of Mycoplasmataceae (Fig. 2b). Subsequently, one MAG of *Mycoplasma, Candidatus* Mycoplasma salmoninae salar (MSS), alone accounted for 83.4% of the host filtered reads. Our analysis revealed a stable MSS dominance across recorded environmental factors, suggesting an ecological adaptation of high-abundance *Mycoplasma* (Suppl. Figs. S4, S5). The richness of *Mycoplasma* MAGs followed a latitudinal pattern showing a mixture between *Mycoplasma* MAGs (NWS_MAG_00006 and NWS_MAG_00013), where NWS_MAG_00013 became more abundant in northern regions, indicating that a higher richness of *Mycoplasma* species could originate from northern Norway or Russia and that more *Mycoplasma* species are to be discovered. We further analysed TARA Oceans data to investigate the putative origin of *Mycoplasma*. Still, no *Mycoplasma* MAGs were found across the Arctic Ocean, suggesting that MSS was unlikely to be obtained from the ocean during the adult life stages of Atlantic salmon.

Reference-based mapping and taxonomy annotation of metagenomics sequence data revealed eukaryotic gut content consisting of diet or gut microfauna (e.g., tapeworms, nematodes, and Myxozoa). Vertebrate diet content in the gut included Atlantic herring (*Clupea harengus*) (Fig. 2c). Gut content of invertebrates was comprised of Arthropoda, Cnidaria, Nematoda, and Platyhelminthes, where arthropods were thought to originate from the diet like krill. Cnidaria, Nematoda, and Platyhelminthes were considered parasitic microfauna, including tapeworms (*Eubothrium*), anisakid nematodes (*Anisakis simplex*), and salmon-related Myxozoan (Fig. 2c). The composition of invertebrates differed according to locations, where cnidarian content dominated the gut content in the south and Platyhelminthes was the dominating phylum in the gut content of northern individuals (Fig. 2c). Despite the compositional differentiation of microfauna and diet, no effect on high-abundance *Mycoplasma* (NWS_MAG_00006) was detected.

### Meta-pangenomics reveals high host specificity of *Mycoplasma*

**Fig. 3:**
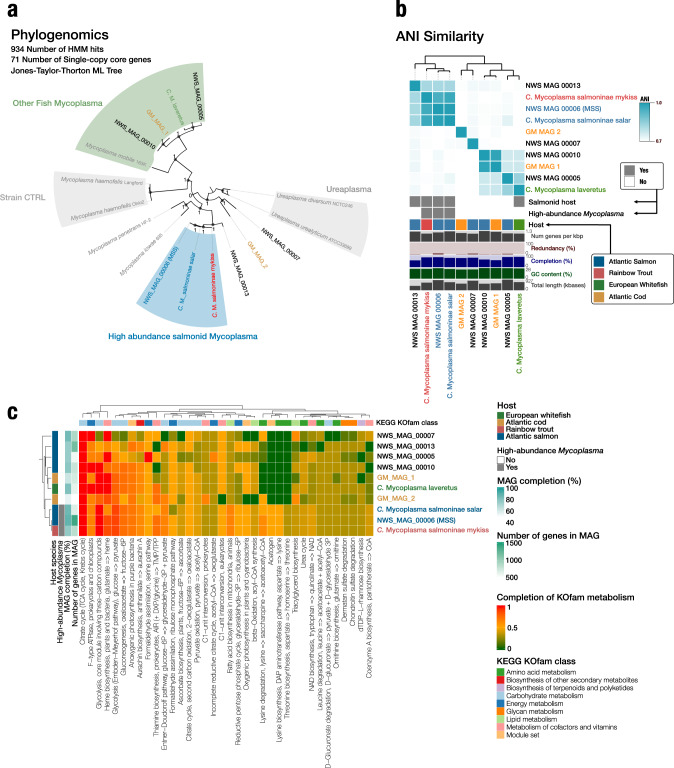
Phylogenomics and comparative genomics of co-assembled MAGs from wild Atlantic salmon and closely related *Mycoplasma* species and candidate genomes. **a** unrooted phylogenomic maximum likelihood tree constructed based on 934 HMM hits from 71 single-copy core genes across 17 genomes and MAGs. **b** Heatmap of Average Nucleotide Identity (ANI) similarity among salmonid-related *Mycoplasma* MAGs from this study and farmed salmonids [15], and *Mycoplasma* MAGs related to Atlantic cod from the same environment [75]. **c** Heatmap of the metabolic reconstruction and comparison of salmonid-related *Mycoplasma* versus other fish-related *Mycoplasma*, based on completion of KEGG pathways.

Our analysis revealed that the high-abundance *Mycoplasma* MAG was closely related to previously recovered salmonid-related *Mycoplasma* MAGs from aquaculture strains (Fig. 3a). Our phylogenomic and comparative analysis revealed that NWS_MAG_00006 and *Candidatus* Mycoplasma salmoninae salar (MSS) were more closely related to each other than the *Mycoplasma, Candidatus* Mycoplasma salmoninae mykiss (MSM), associated with farmed rainbow trout (*Oncorhynchus mykiss*), which underpins the specificity of these salmonid related *Mycoplasma* MAGs to their salmonid host regardless of a farmed or wild origin (Fig. 3a, b). The four other *Mycoplasma-related* MAGs were all found to have low abundance across all individuals and clustered with various salmonid-related clades like NWS_MAG_00013 as a sister group to the clade of Atlantic salmon, rainbow trout, and NWS_MAG_00006. We found two new MAGs related to *Candidatus* Mycoplasma lavaretus (ML) and *Mycoplasma mobile* 163 K. Further, we found one MAG (NWS_MAG_00007) associated with *Ureaplasma*. Analysis of the gene clusters among all *Mycoplasma* MAGs further confirmed the differentiation between MAGs from rainbow trout and Atlantic salmon compared to the other MAGs (Fig. 3a, b). These findings reveal a clade of abundant *Mycoplasma* highly prevalent in salmonids and living in coexistence with other low abundant *Mycoplasma* species.

We found that all high-abundance MAGs in the salmonid clade were not only phylogenetically separated from the other *Mycoplasma* MAGs but also exhibited distinct metabolic traits (Fig. 3c). We reconstructed the metabolic pathways across the fish-related *Mycoplasma* MAGs to decipher the potential functional differences between high and low abundant *Mycoplasma* MAGs for comparison with our previously described phylogenetic differentiation. We found KOfams in the high-abundance clade, which could be potentially beneficial for its host, including genes encoding enzymes involved in thiamine (B1 vitamin) biosynthesis (Fig. 3c). Furthermore, genes involved in the metabolism of nicotinamide/niacin (B3 vitamin) and pantothenate (B5 vitamin) were also found in the high-abundance *Mycoplasma* MAGs (Fig. 3c, Fig. Suppl. S6). Synthesis of two essential amino acids for Atlantic salmon (lysine and threonine), suggesting a potential beneficial role of MSS to its salmon host. Furthermore, we found that the high-abundance clade could function by an acetogenic lifestyle, a function lacking in the other fish-related *Mycoplasma* MAGs. Together, these observations follow a mutualistic relationship where MSS provides this essential feature of acetate fermentation in the predominantly anoxic gut ecosystem of salmonid hosts (Fig. 3c). However, it remains to be shown whether this directly affects the fitness of the salmon host.

### Mirrored patterns of population structures show co-diversification of Atlantic salmon and *Candidatus* Mycoplasma salmoninae salar

To study the evolutionary relationship of wild Atlantic salmon across our sampled locations, we compared our data with Atlantic salmon genotypes of already known origins across north- and southern Norway from publicly available genomes [12]. Principal component analysis (PCA) of host genotype likelihoods revealed three clusters explained by PC1 and PC2 (Fig. Suppl. S7). These clusters were mainly defined by latitude consistent with previous work [11, 12]. Our PCA resulted in three clusters: a cluster (Cluster 1), where the investigated individuals and known individuals were from northern Norway. A cluster (Cluster 2) where the investigated individuals together with known individuals were from southern Norway. Lastly, a cluster with known individuals from the Baltic population. Of the individuals in our data, we detected 24 (32%) individuals from Norwegian populations clustered by latitude. Our analysis revealed that far from all individuals were closely related to the Norwegian genotypes, indicating that several foraging individuals were from genotypes originally reported outside of Norway.

**Fig. 4:**
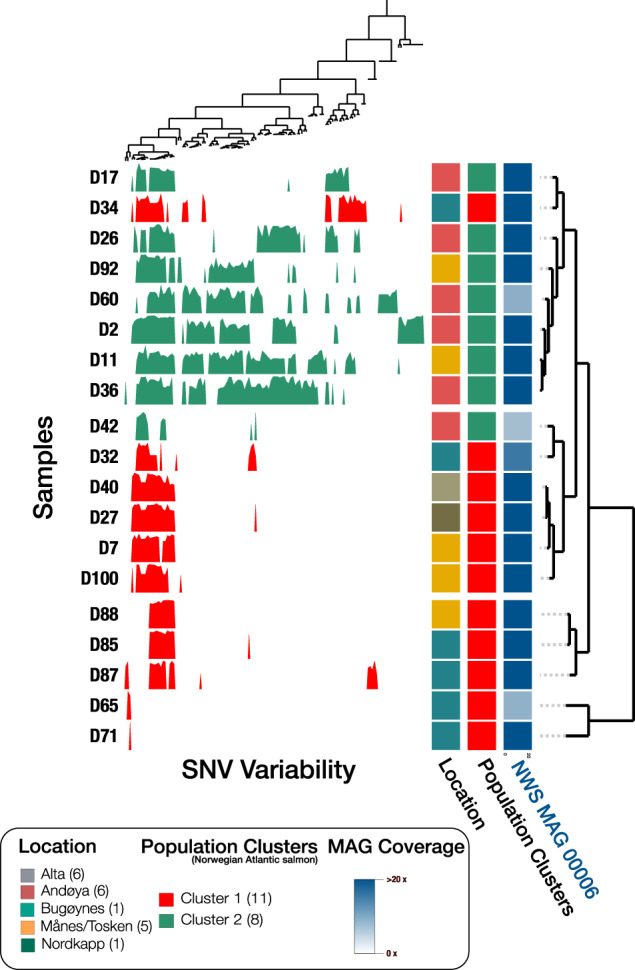
Analysis of host genotypes in Norwegian Atlantic salmon (Host origin) and SNV variability from high-abundance *Mycoplasma* (*Candidatus* Mycoplasma salmoninae salar). Variability of Single Nucleotide Variants (SNVs in the high-abundance *Mycoplasma*) ordered along the *x*-axis. Only SNVs with a minimum of 20X coverage in at least 90% of all individual metagenomes were considered. Each layer represents an individual MSS genotype recovered from a host metagenome. Layers have been hierarchically clustered (dendrogram) based on the similarity of SNV variability (departure from consensus) in MSS between samples using euclidean distance and ward ordination. For each layer, bars for each SNV illustrate variability from zero to one between competing nucleotides (NTs). The SNV variability across samples has been hierarchical clustered (dendrogram) on the *x*-axis, using euclidean distance and ward ordination. The right bar plots represent the host genotype clusters, location, and coverage of MSS. The location information is coloured as indicated by the legend.

Based on their genotypes, these Norwegian lineages were used to infer the possible co-phylogeny between MSS and Atlantic salmon. We chose only to use the Norwegian individuals since the population structure was better described for these individuals. Of the 24 Norwegian individuals, we recovered single nucleotide variants (SNVs) from the associated MSS genomes of 19 individuals (Fig. 4). Analysing SNVs in MSS revealed a total of 136 SNVs present in 90% of the 19 individual metagenomes. Analysis of the Norwegian salmon genotype and MSS showed co-clustering between host populations of Atlantic salmon and the variability of single nucleotide variants (SNVs) in MSS, further supporting co-diversification between MSS and Atlantic salmon. The clustering of MSS was primarily driven by the presence of high SNV variability in Cluster 2, which was absent in Cluster 1, indicating that either Cluster 1 lost its variants or Cluster 2 acquired new variants (Fig. 4). Overall, the mirrored population structure between MSS and Norwegian Atlantic salmon and the constant domination of MSS across any measured environmental factor suggest a high selection pressure on the microbiota. We additionally evaluated patterns of co-diversification with the Parafit analysis, a permutational method that assesses the similarity of principal coordinates derived from the host and the phylogeny of a specific symbiont. The global Parafit test was significant (Parafit, *p*  <  0.001), confirming our findings of co-diversification between Atlantic salmon and MSS.

We looked for evidence that the salmonid MSS population had been subjected to high selection pressure by calculating the average polymorphism rates of non-synonymous to synonymous mutations (pN/pS) for each gene within the MSS population. The genes with pN/pS values higher than one suggested that MSS individuals had been subjected to positive selection pressure (Suppl. Fig.  S8). Most genes with a high pN/pS ratio were unknown. Still, this group also included genes involved in defence mechanisms, translation, ribosomal structure and biogenesis that may have been involved in the adaptation of MSS to the intestinal environment of its salmon host.

**Fig. 5:**
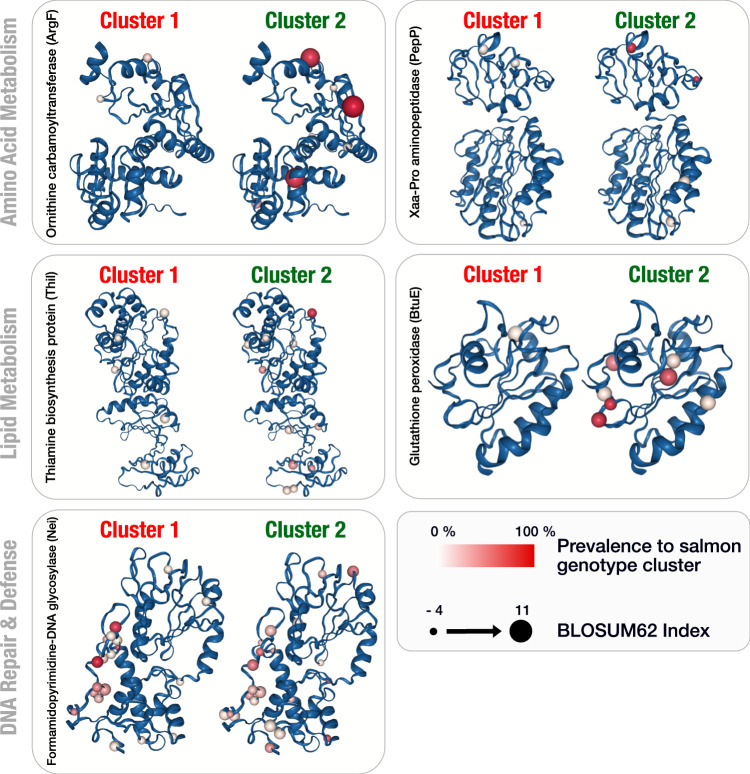
Examples of predicted protein structure variants in *Candidatus* Mycoplasma salmoninae salar related to the host genotype. Single amino acid variants (SAAVs) on the predicted protein structures of five example genes from MSS across 19 metagenomes from the two Atlantic salmon genotypic clusters. The sphere in each predicted protein structure illustrates a SAAV, where colour indicates prevalence concerning the specific host genotype and size of the sphere indicates the interchangeability of two amino acids using BLOcks SUbstitution Matrix (BLOSUM62 index) as noted in the legend.

We also inferred single amino acid variants (SAAVs) to characterise non-synonymous mutations between MSS variants and thereby investigate the putative phenotypic variation in MSS prevalent to its host genotype. Before exploring SAAVs, we predicted protein structures from 141 genes containing SNVs resulting in 51 (36.2%) predicted protein structures with 1813 SAAVs across all individuals. Of the 141 genes containing SNVs, we recovered 51 predicted proteins from the MSS genome. Several of the predicted proteins included SAAVs that were highly prevalent in the related genotype cluster of host salmon (Fig. 5). Three predicted proteins were outstanding in prevalence and relation to Atlantic salmon and the intestinal environment. These proteins included i) ornithine carbamoyl transferase, which is central to the urea cycle and arginine biosynthesis, ii) glutathione peroxidase, involved in peroxide-related defence mechanism in intestinal environments in response to lipid peroxidation [23], and iii) thiamine biosynthesis protein, related to cofactor functions important for lipid metabolism. The SAAVs in these MSS encoded proteins showed a high association with the host genotypic pattern, and our results enable us to predict the genotype of Atlantic salmon by the predicted protein structure variation within MSS conforming with co-diversification of not only synonymous mutations but also non-synonymous mutations between Atlantic salmon and the dominating *Mycoplasma*.

## Discussion

Previous investigations of associations between vertebrate hosts and their microbiota in wild-living species, including teleosts, have mainly been covered by amplicon-based approaches with a low taxonomic resolution and limited functional inference [21, 22, 24]. Moving beyond gene amplicon surveys, we provide the first metagenomic description related to the gastrointestinal environment of multiple wild Atlantic salmon ranging in size, observed stomach content, sequenced diet, genotype, and location. We acknowledge that MAG analysis has limitations, including low sensitivity of low abundant organisms and variable reliability of MAG generation. To increase the reliability of our MAGs, we applied binning methods, combining automatic binning and manual curation with anvi’o, and used good practice for the reliable generation of MAGs [25]. To ensure suitable sequencing depth, we estimated the saturation of ORFs across our samples and applied co-assembly to increase the microbial data for assemblies. Moving beyond gene amplicon surveys of wild Atlantic salmon gut microbiota, we provide a metagenomic description, giving genomic information for lineages missing a functional context and allowing us to search for strain variation within a large pool of microbial populations associated with salmonid hosts. Our results indicate that investigations of salmonid *Mycoplasma* are more complex than previously anticipated and that 16S rRNA gene investigations at the genus level will often simplify the actual strain-level variation in the microbiota.

Our data confirm a low-diverse and *Mycoplasma*-dominated gut microbiota in the Atlantic salmon [13–15]. Furthermore, our analysis also found abundant members of Proteobacteria, including *Aliivibrio*, *Photobacterium*, and *Vibrio*, previously known as part of the gut microbiota in salmonids [26–28]. The microbial variation was low across all 75 investigated individuals despite being exposed to different conditions for multiple environmental factors, indicating high host selection pressure favouring a high relative abundance of *Mycoplasma*. Therefore, we hypothesise that this *Mycoplasma*-dominated microbiota is characteristic of natural and undisturbed Atlantic salmon. Thus, our data should serve as a valuable resource for future microbiota studies in salmonids, including aquaculture applications where gut microbiota investigations have been a research field of increasing interest [26–32].

While numerous cases of co-adaptation have been documented in mammals [33–36], other warm-blooded organisms such as vultures [37], and invertebrates [38, 39], very little is known about the adaptive potential of the associated microbiota in teleosts representing more than half of all vertebrate species. Despite the detailed environmental and life-history metadata for each sampled individual in our study, the environment explained no concluding variation in the abundance of MSS. We found that the SNVs variation of MSS was better explained by the genetic background of the host individual rather than the sampling location, indicating an intriguing host-bacteria co-diversification.

Groussin et al. recently formulated a guideline model to separate co-phylogeny from co-evolution by rejecting the geographic isolation of two host populations [2]. While demonstrations of co-evolution in mammalian and bacterial genomes appear to be demanding [2, 35], our study illustrates that salmon can serve as a valuable future model for expanding our understanding of co-evolution between vertebrate host species and associated bacteria. Indeed, according to Groussin et al., three independent prerequisites are needed for co-evolution to occur with high stability, including i) strong fitness dependencies, ii) stable transmission across generations and iii) strong host selectivity [2]. First, for mammals, they argue that it is non-trivial to convincingly show a direct fitness of symbiotic microbe on its host as the same function can easily be provided by other related species. However, we argue that this is less likely in salmonids due to the low diversity of the microbiota and the low biomass observed. Second, the phylogenomic observation that *Mycoplasma* strains cluster according to both host species, despite being farmed or wild, and population clusters strongly suggest vertical transmission across generations. However, the exact mechanism remains to be elucidated. Though evidence for vertical transmission has not yet been established, other observations besides our study indicate such transmissions, such as the lack of *Mycoplasma* in the surrounding rivers [17] or the Arctic oceans and that *Mycoplasma* species are highly host-dependent due to their intracellular lifestyle. Furthermore, it should be noted that examples of co-evolution do exist based on horizontal transfer, like the bobtail squid-*Vibrio* symbiosis [40] and bean bug-*Burkholdiera* symbiosis [41]. These examples indicate that the evolution of mutualism and the emergence of co-phylogenetic patterns do not strictly rely on strict vertical inheritance [42], but rather a strict host control [43]. Third, our results are in line with previous observations that independently showed positive correlations between *Mycoplasma* abundance and fitness of the salmonid host following strong host selectivity on *Mycoplasma* abundance [26, 44–47]. This is following recent findings showing that *Mycoplasma* abundance does not follow a neutral model in Atlantic salmon, supporting a non-neutral explanation for the observed co-diversification [16]. Our SNV analysis revealed that Cluster 2 of MSS had acquired a higher amount of variable SNV compared to Cluster 1, which is driving the mirrored separation between MSS and Atlantic salmon. Some of these SNVs might come from ecological processes. Overall, our results follow established expectations for co-evolution, presenting salmon as a new model for understanding holobiont evolution [2, 3]. However, our study could not show evidence for reciprocal selection and resulting change. Therefore, we cannot rule out that the co-diversification pattern of MSS and Atlantic salmon could originate from more neutral processes, including ecological processes and genetic drift within the populations of MSS and Atlantic salmon.

Our data provide additional insights into the dominance of *Mycoplasma* and the overall gut microbiota of Atlantic salmon by revealing that several novel, lowly abundant, and dynamic MAGs of *Mycoplasma* co-exist with high-abundance *Mycoplasma*. Furthermore, our data show a clear phylogenomic and functional differentiation between *Mycoplasma* clades of high and low abundance, which will be difficult to decipher using an amplicon-based approach. Specifically, we found a higher completion of pathways associated with the biosynthesis of essential amino acids (lysine and threonine) and biosynthesis of thiamine (B1 vitamin). Wild Atlantic salmon may be deficient in B1 vitamins, suggesting that MSS could be a beneficial source of B1 vitamins in Atlantic salmon [48]. A recent study revealed that the Baltic population of Atlantic salmon lacked the presence of *Mycoplasma*, a population also shown to suffer from B1 vitamin deficiency during early life stages [49] and thereby thiamine deficiency-related reproduction [50–52].

Using untargeted metabolomics, we previously found a higher amount of B vitamins in rainbow trout associated with an increased presence of *C*. Mycoplasma salmoninae mykiss, further supporting some fitness dependency of *Mycoplasma* to its salmonid host [53]. Furthermore, we analysed SAAVs with a high prevalence to host genotype, indicating an apparent fixation of mutations within the MSS populations related to their host. Specifically, we did find SAAVs coupled to thiamine biosynthesis protein and glutathione peroxidase, which were prevalent in the same host population cluster. Both proteins are related to lipid peroxidase, indicating that MSS might be important for lipid metabolism in salmonids or that the diet is important for Atlantic salmon and *Mycoplasma* adaptions. Both scenarios could align with a previous report that revealed alterations in lipid efficiency when *Mycoplasma* was missing in the gut microbiota [53]. In addition, we found SAAVs related to the host in the ornithine carbamoyl transferase protein structure. This protein utilises ammonia and has previously been associated with *Mycoplasma*, indicating a putative adaptation in the intestinal environment [15, 53].

Understanding the functional interdependence between hosts and their microbiota by studying holobionts represents a rewarding field in evolutionary biology [3, 54, 55]. The case of Atlantic salmon studied here has not only furthered our evolutionary understanding of this species, but the findings also hold potential for further discoveries towards feed or health optimisation resulting in more sustainable aquaculture practices. We envisage that our study may inspire similar investigations in systems previously investigated using amplicon-based markers to reveal the intriguing functional host-microbiota interactions.

## Methods

### Ethical approval

Atlantic salmon included in this study were sacrificed immediately upon the catch resulting in instant death before tissue and gut samples were taken. While no separate licence is required for such sampling, we emphasise that all fish handling was supervised by experienced and trained staff following standard and legal procedures in Norway.

### Sample collection

Sample collections for Atlantic salmon were taken from five locations across the coast of northern Norway, including Månes/Torsken, Nordkapp, Alta, Bugøynes, and Andøya. Commercial fishermen caught the Atlantic salmon with nets or traps in coastal waters, as previously described [56–58]. We froze individuals whole immediately after the catch. Sample collections of gut content and gut scrapings were carried out for midgut and hindgut at the National Institute of Nutrition and Seafood Research (NIFES). Atlantic salmon were thawed overnight. Gut sections were divided, and content was squeezed out of the gut using a sterile scalpel. All samples were snap-frozen with dry ice immediately after sample collection. While sample collection was carried out, stomach content, fish weight, length, nematodes, and tapeworm were noticed for subsequent analysis.

### DNA sequencing

DNA was extracted using ZymoBiomics DNA miniPrep (Zymo Research) following the manufacturer’s protocol. Initial quality control of samples was performed using a Qubit 3.0 fluorometer following the manufacturer’s protocol. The DNA was shipped to Novogene (Cambridge, UK) post-extraction for DNA fragmentation by sonication. Library preparation was carried out using Novogene NGS DNA Library Prep Set (Cat No.PT004). Further quality control included qPCR for quantification. Size distribution was detected using Bioanalyzer 2100 (Agilent), and quantified libraries were pooled and sequenced on a NovaSeq 6000 (Illumina) with a 150 bp paired-end strategy.

### Genome-resolved metagenomics

Raw sequence reads were quality controlled, using FastQC/v0.11.8 to assess filtering and quality steps. Adapters and low-quality reads were removed with AdapterRemoval/v2.2.4, with a quality base of 30 and a minimum length of 50 bases. We removed duplicates, and reads were re-paired to remove singletons using bbmap/v.38.35. We filtered data for the host genome using bwa mem [59] to increase assembly efficiency by reducing eukaryotic contaminants. Subsequently, we applied a reference-based mapping and taxonomy annotation to i) remove unknown eukaryotic contaminants and ii) analyse eukaryotic gut content (diet and microfauna) using MGmapper [60]. Filtered data were co-assembled using MEGAHIT with metagenomic sensitive pre-sets [61]. Assembled contigs were quality assessed with Quast/v.5.02. Filtering for a minimal length of 1000 bases per scaffold was applied. To increase effective metagenomics binning, we used the anvi’o pipeline [62, 63] with automatic binning, using CONCOCT, followed by manual curation with the anvi’o platform. Briefly, i) anvi’o was used to identify genes in the scaffolds using Prodigal/v2.6.3 [64] with default parameters. Subsequently, HMMER/v.3.3 [65] were used to identify genes matching archaeal, protists, and bacterial [66] single-copy core gene (SCGs) collections with hidden Markov models (HMMs). Also, ribosomal RNA-based HMMs were identified using available scripts from https://github.com/tseemann/barrnap. The HMMs of SCGs were used to determine the completeness and redundancy of metagenome-assembled genomes (MAGs); ii) read recruitment of the metagenome to the scaffolds was carried out using BWA/v0.7.1596 (minimum identity of 95%) and samtools [67]. We binned contigs automatically using CONCOCT [68]. Each CONCOCT bin was manually curated using the anvi’o interactive interface to ensure high completion and low redundancy. The interface considers each scaffold’s sequence composition, differential coverage, GC content, and taxonomic signal [62, 69]. We defined all bins with >50 % completeness as MAGs. Calculating the potential number of genomes is based on counts of SCGs, following the tutorial outlined at https://merenlab.org/2015/12/07/predicting-number-of-genomes/.

### Identification, refinement, taxonomic, and functional inference of MAGs

We used anvi’o to infer the taxonomy of MAGs based on the proximity of single-copy gene markers based on the Genome Taxonomy Database (GTDB) [70]. Subsequently, we applied Kaiju [71] with NCBI’s non-redundant protein database ‘nr’ to infer the taxonomy of genes (as described in http://merenlab.org/2016/06/18/importing-taxonomy/). For functional inference, we used clusters of orthologous (COGs) [72], Kyoto encyclopedia of genes and genomes (KEGG) [73], and protein families (Pfam) [74], which were annotated through the anvi’o platform. A summary of the MAGs generated for this study is available at 10.6084/m9.figshare.20043452.

### Phylogenomic and comparative analysis of MAGs

Mycoplasma genomes from different species were selected based on relatedness to MAGs from this study. Selected genomes are referred to as external genomes. These external genomes were compared with wild salmonid MAGs using anvi’o/v7.1 for phylogenomic and comparative analysis. The phylogenomic analysis was carried out for all MAGs using an in-house database of 3207 bacterial genomes. Subsequently, we carried out a phylogenomic analysis for *Mycoplasma*, explicitly using the recovered MAGs from this study and MAGs recovered from other studies of salmonids [74] and Atlantic cod (*Gadus morhua*) [75].

Furthermore, outgroups of *Mycoplasma Haemofelis* Langford, *Mycoplasma Haemofelis* Ohio, *Ureaplasma diversum*, and *Ureaplasma urealyticum* ATCC33699 were included. The phylogenomic analysis was carried out based on bacterial SCGs using an anvi’o bacterial database. Amino acid sequences were extracted from HMMs of SCGs and concatenated into aligned amino acid sequences. Concatenated amino acid sequences were used to generate a Newick-based maximum-likelihood phylogeny using FastTree2 [76]. The comparative analysis was carried out as in the previous studies [76], where similarities of each amino acid sequence in every genome were calculated against every other amino acid sequence across all genomes using BLASTp. We implemented Minbit heuristics of 0.5 to eliminate weak matches between two amino acid sequences [77] and an MCL inflation of 2. We used the MCL algorithms to identify gene clusters in amino acid sequence similarity [78]. We calculated ANI using PyANI [79]. Euclidean distance and ward linkage were used to organise gene clusters and genomes. A summary of the pan-genome generated for this study is available at 10.6084/m9.figshare.21183415.

Metabolic reconstruction of compared MAGs was based on KOfams and was carried out using the anvi’o platform. We calculated the level of completeness for a given KEGG module [80, 81] in our genomes using the programme anvi-estimate-metabolism, which leveraged the previous annotation of genes with KEGG orthologs (KOs). The URL https://merenlab.org/m/anvi-estimate-metabolism serves as a tutorial for this programme which details the modes of usage and output file formats. The Heatmap of completion scores was illustrated using the ComplexHeatmap [82] package for R. MAGs were clustered based on similarity across the completion of pathways.

The statistical approach for enrichment analysis is previously defined [83]. Briefly, the programme anvi-compute-functional-enrichment determined enrichment scores for KOfams genomes of low- and high-abundance *Mycoplasma* by fitting a binomial generalised linear model (GLM) to the occurrence of each KOfam in each group and then computing a Rao test statistic. We considered any KOfam with a q-value less than 0.05 to be ‘enriched’ in its associated group. The volcano plot was visualised using the EnhancedVolcano package for R.

### Presence of *Candidatus* Mycoplasma salmoninae salar in the Arctic Ocean

We searched for any detected Tenericutes (a phylum of *Mycoplasma*) across the TARA Ocean data to investigate the presence of *Mycoplasmas* in the Arctic Ocean with an eye toward *Candidatus* Mycoplasma salmoninae salar, using Supplementary information (Table S2) from Delmont et al., which holds the taxonomic information of 1887 recovered MAGs from the global ocean [84].

### Population genetics of *Candidatus* Mycoplasma salmoninae salar

We use the term population to describe an assemblage of co-existing microbial genomes in an environment that can map to the context of the same reference genome [85]. SNVs, single codon variants (SCVs), and SAAVs were used to infer synonymous and non-synonymous mutations in the populations of *C*. Mycoplasma salmoninae salar.

The use of SNVs was related to interest in single nucleotide positions or non-coding regions, whereas SCVs were applied to resolve codon variants, synonymity, and calling of pNpS sites. Lastly, we analysed the structural differences in the encoded proteins by inferring SAAVs. Analyses of SNVs, SCVs, and SAAVs were conducted using anvi’o/7.1, following the tutorial: https://merenlab.org/2015/07/20/analyzing-variability/. Briefly, to study the extent of variation of the MSS genes across all metagenomes, we instructed anvi’o to report positions with more than 1% variation at the nucleotide level (i.e., at least 1% of recruited reads differ from the consensus nucleotide). Here we only reported SNVs with a variation across 90% of the metagenomes to minimise zero inflation. For SCV and SAAV, the programme ‘anvi-gen-variability-profile‘ (with an additional ‘-engine AA’ flag and ‘-engine SCV’ flag) reported variability tables describing the allele frequencies for each SAAV and SCV. Anvi’o only considers short reads that cover the entire codon to determine amino acid frequencies at a given codon position in a metagenome, as previously described [85]. Further inferring of SCVs, pNpS, and SAAVs was based on previous implementations testing for potential selective processes having shaped protein evolution [86]. Prediction of protein structures was carried out with MODELLER [87, 88], embedded in the anvi’o platform. Visualisation of SAAV on predicted protein structures was based on the anvi’o programme “anvi-display-structure” using the following tutorial: https://merenlab.org/2018/09/04/getting-started-with-anvio-structure/.

### Inferring Atlantic salmon population structure

All host-related data were remapped to the Ssal_v3.1 Atlantic salmon genome (Genbank accession GCA_905237065.2) using BWA mem. To estimate the origin of our samples, we spiked data from Atlantic salmon originating from southern and northern Norway, as detailed in Supplementary data S1. To assess the population structure of wild Atlantic salmon across Norway, we applied a genotype likelihood approach to handle low and medium-coverage data using ANGSD [89]. For the estimation of genotype likelihoods, we discarded reads with mapping quality below 20 and bases with base quality below 30.

For the PCA analysis, we used PCAngsd based on genotype likelihoods from variable sites [90]. We filtered genotypic sites for all population structure measurements to minimise false positives related to low coverage and varying sequencing depth across genomes. Filters included filtering for polymorphic sites with a SNP *p* value below 1e-6 and a minimum major/minor allele frequency of 0.05. Furthermore, genotypic sites should be present in at least 50% of the investigated individuals. For clustering of host genotypes, we based our approach on a previous latitudinal description, where the salmon in Norway originate from two main genetic lineages. One lineage from the Barents–White Sea refugium that recolonised northern Norwegian and adjacent Russian rivers, and one from the eastern Atlantic that recolonised the southern and western Norway [11, 12]. The division of the populations into two groups was based on their origin related to Målselva, which was used as a proxy for the middle of the transition zone between populations.

### Statistical test for host-symbiont co-diversification

Parafit [91] was performed based on the Atlantic salmon distance matrix and MSS SNV distance matrix. The MSS SNV distance matrix was based on the SNVs occurring in MSS. The presence/absence of SNV among MSS from different host individuals was used for host-symbiont linkage. The Cailliez correction for negative eigenvalues was applied for the Parafit test [92], and a total of thousand permutations were used.

### World maps

We used the tidyverse [93] and the ggplot2 [94] packages for R to visualise the metagenomic data sets, eukaryotic gut compositions, and relative distribution of MAGs in the world map.

### Statistical analyses

Rarefaction curves were estimated using the *r-package vegan* [95] to infer suitable sequencing depth. The abundance of MAGs was based on mean coverage across each MAG. Data were normalised based on MAG length and were sum-normalised before analysis of ecological dynamics based on TMP normalisation, using the *r-package ADImpute* [96]. Microbial composition analysis was carried out using the *r-package phyloseq* [97]. Correlated response models were used to infer the variation of each MAG related to environmental factors using the *r-package BORAL* [98] as previously described in ref. [44]. Statistical assumptions for parametric and non-parametric analyses were tested using Levene’s test for homogeneity and the Shapiro-Wilk test for normality of data.

## Supplementary information

Supplementary information

Supplemental text and figures

## Data Availability

The raw dataset generated during the current study is available in the European Nucleotide Archive (ENA) repository with project accession number PRJEB59886. Data for comparing Atlantic cod metagenomes are available in the ENA repository with project accession number PRJEB29346. Furthermore, data for estimating the origin of Atlantic salmon populations are based on a previous study [12], which is available in the ENA repository with project accession number PRJEB38061.

## Code availability

The code used for this study is available at: https://github.com/JacobAgerbo/Norwegian_wild_Salmon_2022.
