# Supplementary material for: Co-diversification of an intestinal Mycoplasma and its salmonid host
Source: ISME J. 2023 Feb 17;17(5):682–92. doi: 10.1038/s41396-023-01379-z (PMC10119124; doi:10.1038/s41396-023-01379-z)
Supplement: Supplementary file 1 — Supplementary information [file 41396_2023_1379_MOESM1_ESM.docx]

**Supplementary Information**

Co-diversification of an intestinal *Mycoplasma* and its salmonid host

**Authors:**

Jacob Agerbo Rasmussen (1,2) *

Pia Kiilerich (3)

Abdullah S. Madhun (4)

Rune Waagbø (4) §

Erik-Jan Robert Lock (4)

Lise Madsen (4)

M. Thomas P. Gilbert (2,5)

Karsten Kristiansen (1,6)

Morten Tønsberg Limborg (2) *

* Corresponding authors

§ Rune Waagbø sadly passed away during the final stages of this publication

**Author information:**

1. Laboratory of Genomics and Molecular Medicine, Department of Biology, University of Copenhagen, Copenhagen, Denmark
2. Center for Evolutionary Hologenomics, GLOBE Institute, Faculty of Health and Medical Sciences, University of Copenhagen, Denmark
3. Danish Center for Neonatal Screening, Department of Congenital Disorders, Statens Serum Institut, 2300 Copenhagen, Denmark
4. Institute of Marine Research, Bergen, Norway
5. Department of Natural History, NTNU University Museum, Norwegian University of Science and Technology (NTNU), Trondheim, Norway
6. Institute of Metagenomics, Qingdao-Europe Advanced Institute for Life Sciences, Qingdao China

**Tags:** Comparative genomics, Host adaptation, Mycoplasma, Salmonids, Hologenomic evolution

## Quality Overview of sequencing data

### Table S1) Metagenomic sequencing output and processing

|  | **READS (MILLIONS)** | | | **PERCENTAGE** | |
| --- | --- | --- | --- | --- | --- |
|  | **Raw** | **Mapped to host** | **Unmapped (Bacteria)** | **Mapped to host** | **Unmapped (Bacteria)** |
| **Mean** | 66.35 | 35.40 | 27.90 | 53.82% | 40.74% |
| **Minimum** | 0.52 | 0.31 | 0.01 | 6.84% | 0.10% |
| **Maximum** | 108.93 | 100.12 | 73.39 | 99.74% | 93.75% |
| **SD** | 24.96 | 25.44 | 20.71 | 27.72% | 27.85% |

## Remapping of eukaryotic gut content post host filtering

### Figure S1) Overview of data quantities before and after eukaryotic mapping, using MGmapper. A) Box plots of mapped reads (percentage) to each database, including vertebrates (not mammals), human, vertebrates, invertebrate, and fungi. B) Bar plot illustrating the number of reads before mapping to the MGmapper database (notPhiX) and after extracting all database-related reads, indicating bacterial reads (Unmapped). The black dashed line indicates the mean of unmapped reads, whereas the grey dashed lines indicate the upper and lower 25% quantiles of unmapped reads.

| **MAGs** | **Domain** | **Phylum** | **Class** | **Order** | **Family** | **Genus** | **Species** | **Total Length (bases)** | **No. Contigs** | **N50** | **GC %** | **Completion (%)** | **Redundancy (%)** |
| --- | --- | --- | --- | --- | --- | --- | --- | --- | --- | --- | --- | --- | --- |
| NWS_MAG_00001 | Bacteria | Proteobacteria | Gammaproteobacteria | Enterobacterales | Shewanellaceae | *Shewanella* | Unknown | 3267334 | 473 | 9330 | 38.3 | 98.59 | 1.41 |
| NWS_MAG_00002 | Bacteria | Spirochaetota | Spirochaetia | Brachyspirales | Brachyspiraceae | *Brachyspira* | Unknown | 1326602 | 242 | 7299 | 28.9 | 88.73 | 1.41 |
| NWS_MAG_00003 | Bacteria | Spirochaetota | Spirochaetia | Brachyspirales | Brachyspiraceae | *Brachyspira* | Unknown | 1218193 | 23 | 172397 | 32.7 | 84.51 | 0.00 |
| NWS_MAG_00004 | Bacteria | Proteobacteria | Gammaproteobacteria | Enterobacterales | Shewanellaceae | *Shewanella* | *Shewanella hanedai* | 2871197 | 204 | 18227 | 43.0 | 87.32 | 5.63 |
| NWS_MAG_00005 | Bacteria | Tenericutes | Mollicutes | Mycoplasmatales | Mycoplasmataceae | *Mycoplasma* | Unknown | 927026 | 88 | 17603 | 27.4 | 84.51 | 4.23 |
| NWS_MAG_00006 | Bacteria | Tenericutes | Mollicutes | Mycoplasmatales | Mycoplasmataceae | *Mycoplasma* | Candidatus Mycoplasma salmoninae salar | 876389 | 185 | 9066 | 28.2 | 83.10 | 7.04 |
| NWS_MAG_00007 | Bacteria | Tenericutes | Mollicutes | Mycoplasmatales | Mycoplasmataceae | *Mycoplasma* | Unknown | 826903 | 103 | 14652 | 23.6 | 78.87 | 5.63 |
| NWS_MAG_00008 | Bacteria | Tenericutes | Mollicutes | Mycoplasmatales | Mycoplasmataceae | *Spiroplasma* | Unknown | 936802 | 498 | 1887 | 29.1 | 76.06 | 9.86 |
| NWS_MAG_00009 | Bacteria | Fusobacteriota | Fusobacteriia | Fusobacteriales | Fusobacteriaceae | *Fusobacterium* | Unknown | 700445 | 403 | 1760 | 28.7 | 67.61 | 2.82 |
| NWS_MAG_00010 | Bacteria | Tenericutes | Mollicutes | Mycoplasmatales | Mycoplasmataceae | *Mycoplasma* | Unknown | 562887 | 248 | 2562 | 27.4 | 63.38 | 1.41 |
| NWS_MAG_00011 | Bacteria | Cyanobacteria | Cyanobacteriia | PCC-6307 | Cyanobiaceae | *Synechococcus* | Unknown | 2160500 | 1214 | 1799 | 53.6 | 88.73 | 26.76 |
| NWS_MAG_00012 | Bacteria | Proteobacteria | Gammaproteobacteria | Enterobacterales | Vibrionaceae | *Aliivibrio* | *Aliivibrio salmonicida* | 3227825 | 160 | 23859 | 39.1 | 63.38 | 2.82 |
| NWS_MAG_00013 | Bacteria | Tenericutes | Mollicutes | Mycoplasmatales | Mycoplasmataceae | *Mycoplasma* | Unknown | 378900 | 219 | 1816 | 25.0 | 56.34 | 1.41 |
| NWS_MAG_00014 | Bacteria | Proteobacteria | Gammaproteobacteria | Enterobacterales | Vibrionaceae | *Vibrio* | Unknown | 7414343 | 3874 | 1968 | 42.0 | 59.15 | 5.63 |
| NWS_MAG_00015 | Archaea | Euryarchaeota | Methanococci | Methanococcales | Methanocaldococcaceae | *Methanocaldococcus* | Unknown | 5595641 | 3019 | 1902 | 33.8 | 60.53 | 7.89 |
| NWS_MAG_00016 | Bacteria | Proteobacteria | Gammaproteobacteria | Enterobacterales | Vibrionaceae | *Aliivibrio* | Unknown | 4062199 | 846 | 7080 | 39.2 | 80.28 | 28.17 |
| NWS_MAG_00017 | Bacteria | Proteobacteria | Gammaproteobacteria | Enterobacterales | Vibrionaceae | *Photobacterium* | Unknown | 2730826 | 759 | 5041 | 41.0 | 50.70 | 4.23 |
| NWS_MAG_00018 | Bacteria | Proteobacteria | Gammaproteobacteria | Enterobacterales | Vibrionaceae | *Photobacterium* | *Photobacterium phosphoreum* | 4826908 | 1020 | 7346 | 40.1 | 56.34 | 21.13 |
| NWS_MAG_00019 | Bacteria | Proteobacteria | Gammaproteobacteria | Enterobacterales | Vibrionaceae | *Photobacterium* | Unknown | 6242657 | 1990 | 3798 | 42.0 | 52.11 | 23.94 |
| Unbinned_contigs | NA | NA | NA | NA | NA | NA | NA | 2633685995 | 1121306 | 2711 | 40.8 | 68.67 | 187.95 |

### Table S2) Summary of metagenomic assembled genomes (MAGs)

## Taxonomic inferrence of microbiota in wild Atlantic salmon

### Figure S2) Phylogenomic analysis of wild Atlantic salmon-related Metagenomic Assembled Genomes (MAGs). Unrooted maximum likelihood Phylogenomic tree of 3207 bacteria and the 19 MAGs from the study. Multiple branches without MAGs were collapsed due to illustrative purposes. MAGs are highlighted with red labels, and the closest known taxonomy of all MAGs is labelled with black.

## Analysis of ecological dynamics and effect of environmental factors on gut microbiota

Figure S3) Gut microbiota composition of wild Atlantic salmon. Principal coordinate analysis (PCoA) of recovered MAGs across sampling locations. The PCoA was based on weighted Unifrac distances**.**

Figure S4) Bayesian constrained ordination of environmental factors related to MAGs. A) Constrained ordination of MAGs, where all environmental factors noted in our study are considered. MAGs are labelled orange, whereas samples are labelled **green**. B) Bar plots indicating the proportion of variance explained by known and latent predictors, as indicated in the legend.

### Figure S5) Regression analysis of environmental factors related to MAGs. Correlated response models consist of fitted generalised linear models. Coefficient plots are shown for individual MAGs (along the y-axis) in response to noticed environmental factors as different predictor variables, including putative diet (Arthropoda, Chordata, Mollusca, Echinodermata, Hemichordata), putative parasites (Cnidaria, Ctenophora, Platyhelminthes), observed parasites (nematodes and tapeworm), the latitude of sampling location (as an indicator for sampling location), fitness score of individual fish (Fulton's K factor). NWS_MAG_00006 (*Candidatus* Mycoplasma salmoninae salar) are highlighted in bold blue. Circles in the horizontal bars indicate the coefficient median; filled bars indicate the 95% confidence interval. The shade of green marks the relation between the coefficient, confidence intervals, and null, meaning the darkest green indicates significant correlations (p < 0.05).

###

**Figure S6) Enrichment analysis of specific KOfams.** Volcano plot of 148 KOfams differing between low and high abundant *Mycoplasma* MAGs found in this study. First axis describes enrichment score, whereas secondary axis describes log10 adjusted q-values. Colouring of points are described in legend. Only KOfams significantly different between high and low abundant *Mycoplasma* MAGs were labelled with KEGG based function.


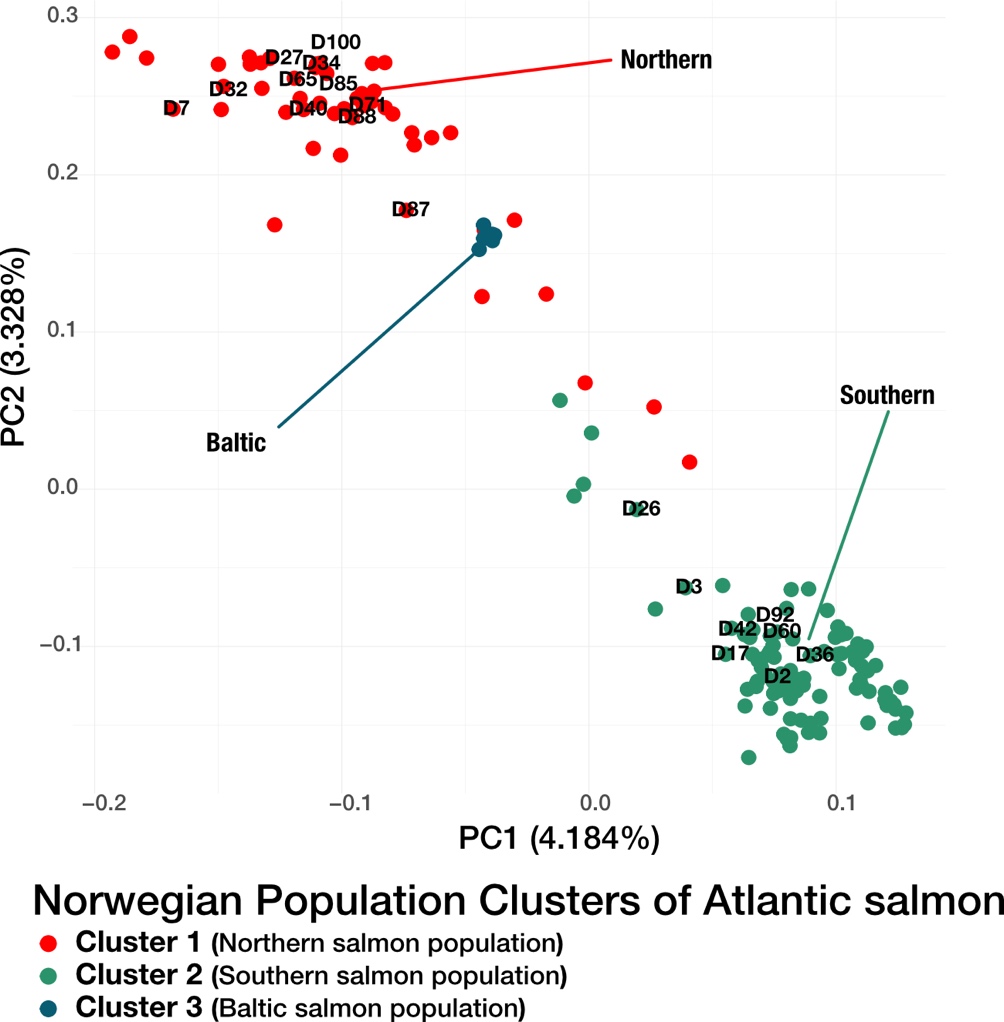


**Figure S7) Host population structure of Atlantic salmon.** Genotype clusters of 206 Norwegian Atlantic salmon, including publicly available genomes and individuals from this study (labelled with black sample names).

###
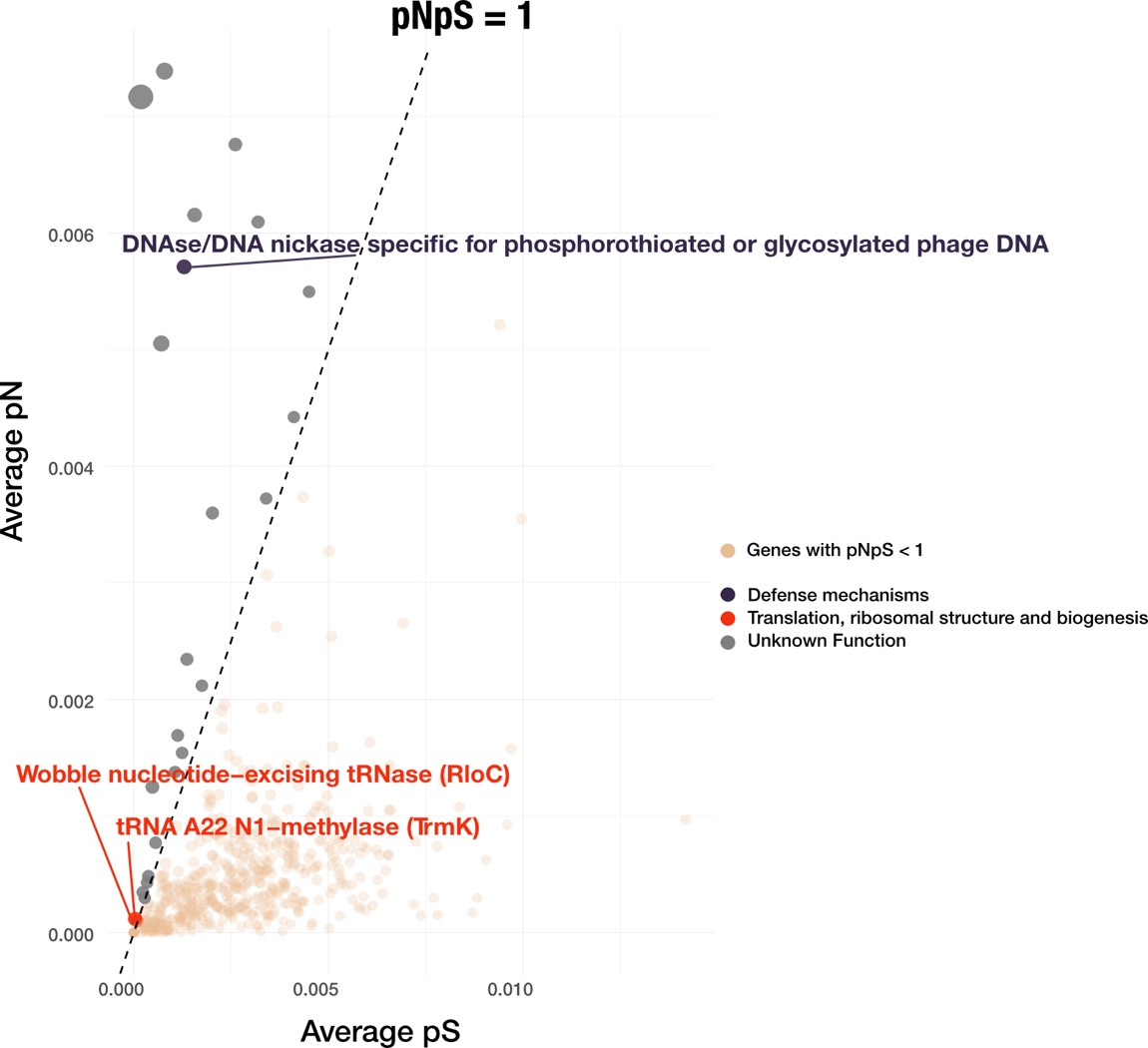


**Figure S8) Non-synonymous and synonymous mutations in *Candidatus* Mycoplasma salmoninae salar.** Scatterplot of pNpS ratio for investigating selection pressure on microbial genes within the MSS MAG. The dashed line indicates a ratio between pN and pS of one. Dot colours indicate functions for genes with a pNpS ratio higher than one, indicating selection, as noted in the legend.
