# Supplementary material for: Co-diversification of an intestinal Mycoplasma and its salmonid host
Source: ISME J. 2023 Feb 17;17(5):682–92. doi: 10.1038/s41396-023-01379-z (PMC10119124; doi:10.1038/s41396-023-01379-z)
Supplement: Supplementary file 2 — Supplemental text and figures [file 41396_2023_1379_MOESM2_ESM.pdf]

Sample	Sampling_Origin	Latitude	Longditude	Clustering	Data	ENA Accession
Alta_12_0038	Alta	69.968	23.375	North	Bertolotti et al.,	PRJEB38061
Alta_12_0111	Alta	69.968	23.375	North	Bertolotti et al.,	PRJEB38061
Alta_12_0135	Alta	69.968	23.375	North	Bertolotti et al.,	PRJEB38061
Arga_12_0015	Aargaardsvassdraget	64.312	11.223	South	Bertolotti et al.,	PRJEB38061
Arga_12_0121	Aargaardsvassdraget	64.312	11.223	South	Bertolotti et al.,	PRJEB38061
Arga_15_0031	Aargaardsvassdraget	64.312	11.223	South	Bertolotti et al.,	PRJEB38061
Arga_15_0201	Aargaardsvassdraget	64.312	11.223	South	Bertolotti et al.,	PRJEB38061
Aroy_12_0084	Aaroey	61.268	7.1671	South	Bertolotti et al.,	PRJEB38061
Aroy_15_0180	Aaroey	61.268	7.1671	South	Bertolotti et al.,	PRJEB38061
Aroy_15_0182	Aaroey	61.268	7.1671	South	Bertolotti et al.,	PRJEB38061
Beia_12_0009	Beiarvassdraget	67.028	14.579	South	Bertolotti et al.,	PRJEB38061
Beia_12_0281	Beiarvassdraget	67.028	14.579	South	Bertolotti et al.,	PRJEB38061
Bors_11_0008	Brselva_i_Porsanger	70.312	25.539	North	Bertolotti et al.,	PRJEB38061
Dale_12_0065	Daleelva_(Hoeyanger)	61.219	6.0748	South	Bertolotti et al.,	PRJEB38061
Driv_12_0056	Driva	62.676064	8.550612	South	Bertolotti et al.,	PRJEB38061
Driv_12_0089	Driva	62.676064	8.550612	South	Bertolotti et al.,	PRJEB38061
Driv_12_0100	Driva	62.676064	8.550612	South	Bertolotti et al.,	PRJEB38061
Eidf_12_0071	Eidfjordvassdraget	60.466	7.0718	South	Bertolotti et al.,	PRJEB38061
Eira_14_48075	Eira	62.678	8.1196	South	Bertolotti et al.,	PRJEB38061
Eira_14_48117	Eira	62.678	8.1196	South	Bertolotti et al.,	PRJEB38061
Eira_14_48228	Eira	62.678	8.1196	South	Bertolotti et al.,	PRJEB38061
Elve_12_0011	Elvegaardselva_(Bjerkvik)	68.546	17.562	South	Bertolotti et al.,	PRJEB38061
Enni_12_0008	Enningdalselva	58.981	11.474	South	Bertolotti et al.,	PRJEB38061
Enni_12_0051	Enningdalselva	58.981	11.474	South	Bertolotti et al.,	PRJEB38061
Flam-15_0008	Flaamselva	60.8651	7.1186	South	Bertolotti et al.,	PRJEB38061
Flek_11_0132	Flekkeelva	61.31	5.3445	South	Bertolotti et al.,	PRJEB38061
Flek_11_0244	Flekkeelva	61.31	5.3445	South	Bertolotti et al.,	PRJEB38061
Fors_12_0038	Forsaavassdraget	68.151	16.116	South	Bertolotti et al.,	PRJEB38061
GauST_13_0058	Gaula_i_Soer-Troendelag	63.341	10.236	South	Bertolotti et al.,	PRJEB38061
GauST_13_0075	Gaula_i_Soer-Troendelag	63.341	10.236	South	Bertolotti et al.,	PRJEB38061
GauST_14_6099	Gaula_i_Soer-Troendelag	63.341	10.236	South	Bertolotti et al.,	PRJEB38061
Glop_11_0001	Gloppenelva	61.768	6.2	South	Bertolotti et al.,	PRJEB38061
Glop_11_0216	Gloppenelva	61.768	6.2	South	Bertolotti et al.,	PRJEB38061
Homl_12_0001	Homla	63.413	10.804	South	Bertolotti et al.,	PRJEB38061
Homl_12_0002	Homla	63.413	10.804	South	Bertolotti et al.,	PRJEB38061
Homl_12_0003	Homla	63.413	10.804	South	Bertolotti et al.,	PRJEB38061
Jols_15_0663	Joelstra	61.455	5.8434	South	Bertolotti et al.,	PRJEB38061
Jols_15_36274	Joelstra	61.455	5.8434	South	Bertolotti et al.,	PRJEB38061
Jols_15_36277	Joelstra	61.455	5.8434	South	Bertolotti et al.,	PRJEB38061
Keret_A12	Keret	66.021367	32.787895	White sea	Bertolotti et al.,	PRJEB38061
Keret_B12	Keret	66.021367	32.787895	White sea	Bertolotti et al.,	PRJEB38061
Keret_H11	Keret	66.021367	32.787895	White sea	Bertolotti et al.,	PRJEB38061
Koma_06_0007	Komagelva	70.242	30.522	North	Bertolotti et al.,	PRJEB38061
Koma_06_0009	Komagelva	70.242	30.522	North	Bertolotti et al.,	PRJEB38061
Koma_06_0010	Komagelva	70.242	30.522	North	Bertolotti et al.,	PRJEB38061
Laer_07_0016	Laerdalselva	61.102	7.4725	South	Bertolotti et al.,	PRJEB38061
Lakj_03_0009	Tana-Laksjohka	70.059	27.562	North	Bertolotti et al.,	PRJEB38061
Lakj_10_0318	Tana-Laksjohka	70.059	27.562	North	Bertolotti et al.,	PRJEB38061
Laks_12_0006	Lakselva_i_Porsanger	70.078	24.927	North	Bertolotti et al.,	PRJEB38061
Laks_12_0106	Lakselva_i_Porsanger	70.078	24.927	North	Bertolotti et al.,	PRJEB38061
LanG_12_0012	Langfjordvassdraget	70.669135	27.839054	North	Bertolotti et al.,	PRJEB38061
LanG_12_0025	Langfjordvassdraget	70.669135	27.839054	North	Bertolotti et al.,	PRJEB38061
LanG_12_0028	Langfjordvassdraget	70.669135	27.839054	North	Bertolotti et al.,	PRJEB38061
Lauk_13_0013	Laukhellevassdraget_(Lakselva_fra_Trollbuvatnet)	69.227	17.849	North	Bertolotti et al.,	PRJEB38061
Lauk_13_0016	Laukhellevassdraget_(Lakselva_fra_Trollbuvatnet)	69.227	17.849	North	Bertolotti et al.,	PRJEB38061
Lauk_13_0017	Laukhellevassdraget_(Lakselva_fra_Trollbuvatnet)	69.227	17.849	North	Bertolotti et al.,	PRJEB38061
Lone_12_0023	Loneelva_i_Osteroey	60.52	5.5011	South	Bertolotti et al.,	PRJEB38061
Mals_11_0025	Maalselvvassdraget	69.264	18.51	North	Bertolotti et al.,	PRJEB38061
Mals_12_0020	Maalselvvassdraget	69.264	18.51	North	Bertolotti et al.,	PRJEB38061
Mals_12_0022	Maalselvvassdraget	69.264	18.51	North	Bertolotti et al.,	PRJEB38061
Mask_06_0199	Tana-Maskejohka	70.285	28.163	North	Bertolotti et al.,	PRJEB38061
Mask_06_0200	Tana-Maskejohka	70.285	28.163	North	Bertolotti et al.,	PRJEB38061
Nams_12_0049	Namsen_(hele_vassdraget)	64.464	11.682	South	Bertolotti et al.,	PRJEB38061
Nams_12_0267	Namsen_(hele_vassdraget)	64.464	11.682	South	Bertolotti et al.,	PRJEB38061
Nams_15_405	Namsen_(hele_vassdraget)	64.464	11.682	South	Bertolotti et al.,	PRJEB38061
Nams_15_494	Namsen_(hele_vassdraget)	64.464	11.682	South	Bertolotti et al.,	PRJEB38061
Naus_12_0016	Nausta	61.506	5.7197	South	Bertolotti et al.,	PRJEB38061
Naus_12_0038	Nausta	61.506	5.7197	South	Bertolotti et al.,	PRJEB38061
Naus_12_0041	Nausta	61.506	5.7197	South	Bertolotti et al.,	PRJEB38061
Neid_13_1181	Neiden	69.701425	29.525231	North	Bertolotti et al.,	PRJEB38061
Neid_13_2888	Neiden	69.701425	29.525231	North	Bertolotti et al.,	PRJEB38061
Neid_13_2899	Neiden	69.701425	29.525231	North	Bertolotti et al.,	PRJEB38061
Nume_12_0015	Numedalslaagen	59.06	10.071	South	Bertolotti et al.,	PRJEB38061
Nume_12_0043	Numedalslaagen	59.06	10.071	South	Bertolotti et al.,	PRJEB38061
Nume_12_0051	Numedalslaagen	59.06	10.071	South	Bertolotti et al.,	PRJEB38061
Orkl_12_0804	Orkla	63.306627	9.82683	South	Bertolotti et al.,	PRJEB38061
Orkl_12_0885	Orkla	63.306627	9.82683	South	Bertolotti et al.,	PRJEB38061
Orkl_12_0899	Orkla	63.306627	9.82683	South	Bertolotti et al.,	PRJEB38061
Osel_11_0055	Oselva_i_Os	60.186	5.4723	South	Bertolotti et al.,	PRJEB38061

Osen_10_0003	Oselvvassdraget_(Osen)	61.550626	5.413458	South	Bertolotti et al.,	PRJEB38061
Osen_10_0015	Oselvvassdraget_(Osen)	61.550626	5.413458	South	Bertolotti et al.,	PRJEB38061
Osen_10_0017	Oselvvassdraget_(Osen)	61.550626	5.413458	South	Bertolotti et al.,	PRJEB38061
Reip_11_0010	Reipaaga	66.908	13.632	South	Bertolotti et al.,	PRJEB38061
Reip_11_0011	Reipaaga	66.908	13.632	South	Bertolotti et al.,	PRJEB38061
Reip_11_0012	Reipaaga	66.908	13.632	South	Bertolotti et al.,	PRJEB38061
Repp_12_0010	Repparfjordelva	70.445	24.328	North	Bertolotti et al.,	PRJEB38061
Repp_12_0011	Repparfjordelva	70.445	24.328	North	Bertolotti et al.,	PRJEB38061
Repp_12_0019	Repparfjordelva	70.445	24.328	North	Bertolotti et al.,	PRJEB38061
Risf_11_0006	Risfjordvassdraget	70.978	28.171	North	Bertolotti et al.,	PRJEB38061
Risf_11_0011	Risfjordvassdraget	70.978	28.171	North	Bertolotti et al.,	PRJEB38061
Risf_11_0015	Risfjordvassdraget	70.978	28.171	North	Bertolotti et al.,	PRJEB38061
Roks_12_0111	Roksdalsvassdraget	69.05	15.869	North	Bertolotti et al.,	PRJEB38061
Roks_12_0113	Roksdalsvassdraget	69.05	15.869	North	Bertolotti et al.,	PRJEB38061
Rygg_12_0002	Ryggelva	61.779	6.1249	South	Bertolotti et al.,	PRJEB38061
Rygg_12_0008	Ryggelva	61.779	6.1249	South	Bertolotti et al.,	PRJEB38061
Rygg_12_0010	Ryggelva	61.779	6.1249	South	Bertolotti et al.,	PRJEB38061
Salt_12_0036	Saltdalsvassdraget	67.098	15.419	South	Bertolotti et al.,	PRJEB38061
Salt_12_0039	Saltdalsvassdraget	67.098	15.419	South	Bertolotti et al.,	PRJEB38061
Salt_12_0041	Saltdalsvassdraget	67.098	15.419	South	Bertolotti et al.,	PRJEB38061
Sand_12_0004	Sandfjordelva_i_Gamvik	71.049	28.057	North	Bertolotti et al.,	PRJEB38061
Sand_12_0006	Sandfjordelva_i_Gamvik	71.049	28.057	North	Bertolotti et al.,	PRJEB38061
Sand_12_0038	Sandfjordelva_i_Gamvik	71.049	28.057	North	Bertolotti et al.,	PRJEB38061
Skie_12_0091	Skienselva	59.135	9.6301	South	Bertolotti et al.,	PRJEB38061
Skip_12_0014	Skipsfjordvassdraget	70.158	19.797	North	Bertolotti et al.,	PRJEB38061
Skip_12_0027	Skipsfjordvassdraget	70.158	19.797	North	Bertolotti et al.,	PRJEB38061
SS_08_MSW_003_D11	Tornio	65.84811	24.14662	Baltic	Bertolotti et al.,	PRJEB38061
SS_08_MSW_007_E11	Tornio	65.84811	24.14662	Baltic	Bertolotti et al.,	PRJEB38061
SS_08_MSW_008_F11	Tornio	65.84811	24.14662	Baltic	Bertolotti et al.,	PRJEB38061
SS_08_MSW_015_B12	Tornio	65.84811	24.14662	Baltic	Bertolotti et al.,	PRJEB38061
SS_08_MSW_016_C12	Tornio	65.84811	24.14662	Baltic	Bertolotti et al.,	PRJEB38061
SS_08_MSW_019_E12	Tornio	65.84811	24.14662	Baltic	Bertolotti et al.,	PRJEB38061
SS_08_MSW_023_F12	Tornio	65.84811	24.14662	Baltic	Bertolotti et al.,	PRJEB38061
Suld_11_0118	Suldalslaagen	59.48	6.2506	South	Bertolotti et al.,	PRJEB38061
Suld_11_0270	Suldalslaagen	59.48	6.2506	South	Bertolotti et al.,	PRJEB38061
Suld_11_0303	Suldalslaagen	62.971	8.6624	South	Bertolotti et al.,	PRJEB38061
Surn_13_0028	Surna	62.971	8.6624	South	Bertolotti et al.,	PRJEB38061
Surn_13_0280	Surna	62.838	7.2096	South	Bertolotti et al.,	PRJEB38061
Sylt_12_0055	Sylteelva_i_Fraena	62.838	7.2096	South	Bertolotti et al.,	PRJEB38061
Sylt_12_0103	Sylteelva_i_Fraena	69.909	27.0285	North	Bertolotti et al.,	PRJEB38061
Vigd_09_0001	Vigda	63.312	10.182	South	Bertolotti et al.,	PRJEB38061
Vigd_09_0004	Vigda	63.312	10.182	South	Bertolotti et al.,	PRJEB38061
Vigd_09_0006	Vigda	63.312	10.182	South	Bertolotti et al.,	PRJEB38061
Vike_12_0013	Vikedalselva_i_Vindafjord	59.496	5.8972	South	Bertolotti et al.,	PRJEB38061
Vike_12_0014	Vikedalselva_i_Vindafjord	59.496	5.8972	South	Bertolotti et al.,	PRJEB38061
Vike_13_0016	Vikedalselva_i_Vindafjord	59.496	5.8972	South	Bertolotti et al.,	PRJEB38061
Vorm_11_0035	Vorma	59.271	6.3322	South	Bertolotti et al.,	PRJEB38061
Vorm_11_0039	Vorma	59.271	6.3322	South	Bertolotti et al.,	PRJEB38061
D100	Moenes/Tosken	Unknown	Unknown	Unknown	This Study	
D109	Bugoeynes	Unknown	Unknown	Unknown	This Study	
D10	Alta	Unknown	Unknown	Unknown	This Study	
D110	Bugoeynes	Unknown	Unknown	Unknown	This Study	
D111	Bugoeynes	Unknown	Unknown	Unknown	This Study	
D112	Bugoeynes	Unknown	Unknown	Unknown	This Study	
D113	Bugoeynes	Unknown	Unknown	Unknown	This Study	
D114	Bugoeynes	Unknown	Unknown	Unknown	This Study	
D11	Moenes/Tosken	Unknown	Unknown	Unknown	This Study	
D12	Andoeya	Unknown	Unknown	Unknown	This Study	
D13	Moenes/Tosken	Unknown	Unknown	Unknown	This Study	
D14	Nordkapp	Unknown	Unknown	Unknown	This Study	
D15	Nordkapp	Unknown	Unknown	Unknown	This Study	
D16	Moenes/Tosken	Unknown	Unknown	Unknown	This Study	
D17	Andoeya	Unknown	Unknown	Unknown	This Study	
D18	Bugoeynes	Unknown	Unknown	Unknown	This Study	
D20	Nordkapp	Unknown	Unknown	Unknown	This Study	
D21	Andoeya	Unknown	Unknown	Unknown	This Study	
D22	Nordkapp	Unknown	Unknown	Unknown	This Study	
D23	Moenes/Tosken	Unknown	Unknown	Unknown	This Study	
D24	Nordkapp	Unknown	Unknown	Unknown	This Study	
D26	Andoeya	Unknown	Unknown	Unknown	This Study	
D27	Nordkapp	Unknown	Unknown	Unknown	This Study	
D2	Andoeya	Unknown	Unknown	Unknown	This Study	
D30	Andoeya	Unknown	Unknown	Unknown	This Study	
D31	Bugoeynes	Unknown	Unknown	Unknown	This Study	
D32	Alta	Unknown	Unknown	Unknown	This Study	
D33	Moenes/Tosken	Unknown	Unknown	Unknown	This Study	
D34	Alta	Unknown	Unknown	Unknown	This Study	
D35	Bugoeynes	Unknown	Unknown	Unknown	This Study	
D36	Andoeya	Unknown	Unknown	Unknown	This Study	
D37	Nordkapp	Unknown	Unknown	Unknown	This Study	

D3	Andoeya	Unknown	Unknown	Unknown	This Study
D40	Bugoeynes	Unknown	Unknown	Unknown	This Study
D42	Andoeya	Unknown	Unknown	Unknown	This Study
D44	Nordkapp	Unknown	Unknown	Unknown	This Study
D45	Alta	Unknown	Unknown	Unknown	This Study
D46	Andoeya	Unknown	Unknown	Unknown	This Study
D47	Bugoeynes	Unknown	Unknown	Unknown	This Study
D4	Nordkapp	Unknown	Unknown	Unknown	This Study
D51	Nordkapp	Unknown	Unknown	Unknown	This Study
D53	Nordkapp	Unknown	Unknown	Unknown	This Study
D54	Andoeya	Unknown	Unknown	Unknown	This Study
D55	Nordkapp	Unknown	Unknown	Unknown	This Study
D60	Andoeya	Unknown	Unknown	Unknown	This Study
D64	Andoeya	Unknown	Unknown	Unknown	This Study
D65	Alta	Unknown	Unknown	Unknown	This Study
D69	Andoeya	Unknown	Unknown	Unknown	This Study
D70	Andoeya	Unknown	Unknown	Unknown	This Study
D71	Alta	Unknown	Unknown	Unknown	This Study
D73	Moenes/Tosken	Unknown	Unknown	Unknown	This Study
D75	Bugoeynes	Unknown	Unknown	Unknown	This Study
D77	Bugoeynes	Unknown	Unknown	Unknown	This Study
D79	Moenes/Tosken	Unknown	Unknown	Unknown	This Study
D7	Moenes/Tosken	Unknown	Unknown	Unknown	This Study
D81	Bugoeynes	Unknown	Unknown	Unknown	This Study
D82	Bugoeynes	Unknown	Unknown	Unknown	This Study
D83	Alta	Unknown	Unknown	Unknown	This Study
D84	Bugoeynes	Unknown	Unknown	Unknown	This Study
D85	Alta	Unknown	Unknown	Unknown	This Study
D87	Alta	Unknown	Unknown	Unknown	This Study
D88	Moenes/Tosken	Unknown	Unknown	Unknown	This Study
D8	Andoeya	Unknown	Unknown	Unknown	This Study
D90	Alta	Unknown	Unknown	Unknown	This Study
D92	Moenes/Tosken	Unknown	Unknown	Unknown	This Study
D93	Alta	Unknown	Unknown	Unknown	This Study
D97	Bugoeynes	Unknown	Unknown	Unknown	This Study
D9	Nordkapp	Unknown	Unknown	Unknown	This Study
MG104	Bugoeynes	Unknown	Unknown	Unknown	This Study
MG38	Alta	Unknown	Unknown	Unknown	This Study
MG41	Nordkapp	Unknown	Unknown	Unknown	This Study
MG48	Nordkapp	Unknown	Unknown	Unknown	This Study
MG54	Andoeya	Unknown	Unknown	Unknown	This Study
MG59	Nordkapp	Unknown	Unknown	Unknown	This Study
MG98	Moenes/Tosken	Unknown	Unknown	Unknown	This Study
MG99	Bugoeynes	Unknown	Unknown	Unknown	This Study
